# Entertainments in Hospitals

**Published:** 1916-01-01

**Authors:** 


### ENTERTAINMENTS IN HOSPITALS.

The Christmas season does not fail to provoke good will in the domestic sphere, even when very different sentiments are prevalent in the international atmosphere. Naturally one expression of it is directed towards the less fortunate members of the community, and among these the sick and suffering collected in the hospitals find an honoured place. Every hospital secretary, not to speak of other members of the staff, is made aware at this time of the year of numbers of people who are anxious to contribute some active evidences of interest in the patients and wish to do something to minister to their happiness. Sympathy with suffering seems to become especially conspicuous, and in the fashion here suggested it takes shape in quarters where it would not always be anticipated. There are this year rather more than fewer evidences of this kindly interest, and, of course, it is heartily welcomed. The war has emphasised the value of the hospitals, and from the part they have played in the present emergency, many people have been led to consider more intelligently their normal function in the social organisation. Hence an extended desire to take some share in their charitable mission.

One proposal which has many supporters is the organisation of musical or kindred entertainments for the patients. These often reach a high level of excellence, and usually give a great deal of pleasure. Occasionally the level may be pitched a little high, but for the most part the programme is characterised both by good judgment and good taste; and everyone having experience of such entertainments knows full well that the patients are quick to appreciate these qualities. The performances give pleasure both to the patients and to the performers, and in a practical world it is necessary to keep both these aspects of the matter in view. It is necessary to say this because the medical staff does not always look with unmixed satisfaction on these invasions of an area in which the doctors have to bear personal responsibility for the welfare of people who admittedly are more or less seriously ill. And it cannot be denied that the results are not invariably pleasing. Raised temperatures and other evidences of constitutional disturbance are not infrequent consequences, and over these the doctors cannot do other than shake their heads. Probably the ideal course would be to confine the entertainments to a ward or room temporarily reserved for the purpose and to bring to this only such patients as can be moved with safety. Yet such a policy would produce much disappointment. It would disappoint many patients, who would feel themselves excluded from what has come to be regarded as a normal part of hospital life at Christmastide. Not less would it disappoint many sympathetic helpers, who would scarcely recognise that their real motive was met by anything short of a hospital ward with its pathetic evidences of human suffering. That a medical veto in individual instances is necessary cannot be contested, but too rigid an insistence on this may hinder a custom which has values both to those who give and to those who receive. No impartial person, at all events, could read with understanding the accounts which we publish of the way Christmas has been celebrated throughout the hospitals of the country without realising how much these entertainments mean to all concerned. As one of the writers puts it, it is not so much what is done as the atmosphere which is created that counts. Its spirit lives in the pages which follow, and the accounts show that the Christmas festivities are no mere ebullience of high spirits, but the spirit of personal service in the disguise of Santa Claus. As Santa Claus himself is the unseen worker from whose generous labour everyone benefits, so he becomes in hospital the actual embodiment of the spirit which is at work in the wards not only at Christmas, but every day throughout the year.

The matter is rather a complicated one, for anyone who goes often round a hospital ward in which Christmas entertainments are proceeding will not fail to meet with a patient or two, whose condition may seem to suggest that he finds the noise and the singing anything but cheering. Yet when asked what are his views on the matter, such a patient is often heard to say that he would not be deprived of the sense of Christmas thus given to him, however little capable he may be of joining in the celebrations himself. The matter, like many others in hospital administration, must be solved on common-sense lines, and this involves the exclusion of the entertainers from certain wards or the removal of certain patients to quiet quarters. The scales in any case have to be held carefully, since the desire to remain in hospital over the festival in the case of patients really fit to be discharged has also to be sympathetically considered, and, indeed, it would probably be found that during Christmas week most hospitals contain a lower proportion of really grave cases than usual. The fact that the entertainments remain permanent features of institutional life at this season, in spite of the annual criticisms to which they are liable, is perhaps sufficient to show that, on the whole, they justify their claim, and fulfil their purpose innocuously.

